# Human Multipotent Mesenchymal Stromal Cell–Derived Extracellular Vesicles Enhance Neuroregeneration in a Rat Model of Sciatic Nerve Crush Injury

**DOI:** 10.3390/ijms23158583

**Published:** 2022-08-02

**Authors:** Svetlana V. Demyanenko, Maria A. Pitinova, Yulia N. Kalyuzhnaya, Andrey M. Khaitin, Svetlana A. Batalshchikova, Natalya M. Dobaeva, Yulia A. Shevtsova, Kirill V. Goryunov, Egor Y. Plotnikov, Svetlana G. Pashkevich, Gennady T. Sukhikh, Denis N. Silachev

**Affiliations:** 1 Laboratory of Molecular Neurobiology, Academy of Biology and Biotechnology, Southern Federal University, pr. Stachki 194/1, 344090 Rostov-on-Don, Russia; brevde@sfedu.ru (M.A.P.); ykalyuzhnaya@sfedu.ru (Y.N.K.); amhaitin@sfedu.ru (A.M.K.); batalshikova@sfedu.ru (S.A.B.); 2 Department of General and Clinical Biochemistry No.2, Rostov State Medical University, St. Nakhichevansky 29, 344000 Rostov-on-Don, Russia; bnm8@mail.ru; 3 V.I. Kulakov National Medical Research Center of Obstetrics, Gynecology and Perinatology, 117997 Moscow, Russia; yu_shevtsova@oparina4.ru (Y.A.S.); k_gorunov@oparina4.ru (K.V.G.); g_sukhikh@oparina4.ru (G.T.S.); 4 Faculty of Bioengineering and Bioinformatics, Lomonosov Moscow State University, 119992 Moscow, Russia; 5 A.N. Belozersky Institute of Physico-Chemical Biology, Lomonosov Moscow State University, 119992 Moscow, Russia; plotnikov@belozersky.msu.ru; 6 State Scientific Institution «Institute of Physiology of the National Academy of Sciences of Belarus», Minsk, Akademicheskaya Str., 28, 220072 Minsk, Belarus; pashkevich@physiology.by

**Keywords:** apoptosis, dorsal root ganglion, nerve injury, regeneration, multipotent mesenchymal cells, extracellular vesicles, neuroprotection

## Abstract

Peripheral nerve injury remains a serious problem for medicine, with no effective method of treatment at the moment. The most prominent example of this problem is neonatal brachial plexus palsy, which results from the stretching of the brachial plexus nerves in the birth or perinatal period. Multipotent mesenchymal cells (MSCs) and the extracellular vesicles (EVs) they produce are known to have a marked neuroprotective effect in central nervous system injuries. We suggested that the use of MSCs-derived EVs may be an effective approach to the regeneration of peripheral nerves after injury. Sciatic nerve injury was modeled in rats via crushing, and then a gel containing MSCs–EVs was applied to the injured area. After 15 and 30 days, a histological, physiological, and functional assessment of nerve, dorsal root ganglia (DRG), and innervated muscles’ recovery was performed. Transplantation of EVs to the area of sciatic nerve injury significantly reduced muscle atrophy as compared to the control group. Functional recovery of the innervated muscles, as measured by the extensor postural thrust test, was revealed 30 days after the surgery. We associate the obtained results with EVs-induced neuroprotective mechanisms, which were expressed in a decrease in apoptotic neuronal death and an increase in regeneration-associated proteins NF-200 and GAP-43, as well as in DRG and damaged nerve. We suggest that the therapeutic scheme we used is efficient for the treatment of acute peripheral nervous system injuries and can be transferred to the clinics. However, additional studies are required for a more detailed analysis of neuroprotection mechanisms.

## 1. Introduction

Peripheral nerve injury remains a serious clinical problem without ultimate solution for now. Despite significant advances in microsurgery, some severe types of nervous system traumas are impossible to cure without causing complications in the damaged nerve [1]. A striking example here is such a birth injury as neonatal brachial plexus palsy (NBPP), occurring as a result of brachial nerve extension in birth or perinatal period, which manifests as a weak or paralyzed upper extremity in a newborn [2]. This pathology affect 0.1 to 8.1 per 1000 newborns all over the world [3]. Such traumas have negative impacts on a child’s physical development, not to mention his or her psycho-emotional condition and the resulting socioeconomical burden on the state [4].

Today, NBPP is curable only by using autogenous nerve grafts (autografts), nerve transfer, tendon transfer, and free functioning muscle transfer [5,6]. However, it is worth noting that these approaches entail a number of risks, such as the presence of undetected infection in donor material, high risk of surgical complications, shortage of transplantation materials, and mismatch of diameters and/or nerve fascicles. Due to the active development of tissue engineering, tissue-engineered nerve transplants are considered a promising prospect [7]. This construct usually consists of a scaffold with abundant supporting cells and/or growth factors [8]. The scaffold provides a matrix for direction and remodeling of damaged axons [9], while growth factors accelerate nerve regeneration [10]. However, there are a number of limitations in using growth factors. For example, the nerve growth factor (NGF) treatment can be inappropriate because of the impossibility of accurate regulation of dosage and directed effect of the growth factor exactly along the way of future axon growth; this results in uncontrolled and undirected axon growth in growing cones [11,12].

In this connection appears a problem of the search for new therapeutical approaches, providing not only regular production of growth factors, but also their dosed delivery to the lesion sited.

In recent years, it has been shown that using multipotent mesenchymal stromal cells (MSCs) is a prospective method for the treatment of damaged nerve fibers [1]. For example, experimental sciatic nerve damage in rats and following perineural transplantation of MSCs from canine adipose tissue to the lesion zone resulted in four weeks in significant functional nerve recovery, with electromyographic indices being close to normal [13].

Many researchers consider the secretion of extracellular vesicles (EVs), not the direct differentiation and functional neural cells, to be the main mechanism of MSCs’ therapeutic effect [14,15]. EVs are a group of heterogeneous nanoparticles which are conventionally distinguished by their size and biogenesis. Thus, particles formed via external budding from the cell membrane (with size from ~0.1 mm to 1 µm) are called ectosomes. Exosomes are another class of cell-derived extracellular vesicles of endosomal origin, typically with diameter of ~30–200 nm [16]. EVs, regardless of their origin, contain various signaling molecules (proteins, lipids, mRNA, miRNA, ncRNA, circRNA, and DNA), which mediate intercellular in the fusion of vesicles with target cells [17,18]. EVs reproduce a range of therapeutic effects of MSCs; in the future, this will allow us to avoid a number of shortcomings and overcome limitations of MSCs transplantation [15,19,20]. As for today, there are just isolated works demonstrating a potential possibility of peripheral nerve repair by using MSCs-derived EVs. Thus, in a model of injury to superior laryngeal nerve, responsible for swallowing, it was demonstrated that the secretome of human milk teeth pulp MSCs recovered the swallowing function and myelinization of injured nerve due to the attraction of anti-inflammatory M2 macrophages to the injury site [21]. Nevertheless, the application of extracellular vesicles for peripheral nerve regeneration seems to be a prospective approach, and the research has to be aimed at studying optimal treatment regimens and regeneration mechanisms [22].

The goal of this work was to study the possibility of nerve fiber regeneration by using extracellular vesicles embedded into collagen gel in a model system of rat sciatic nerve injury.

## 2. Results

### 2.1. Characterization of Extracellular Vesicles

**Figure 1:**
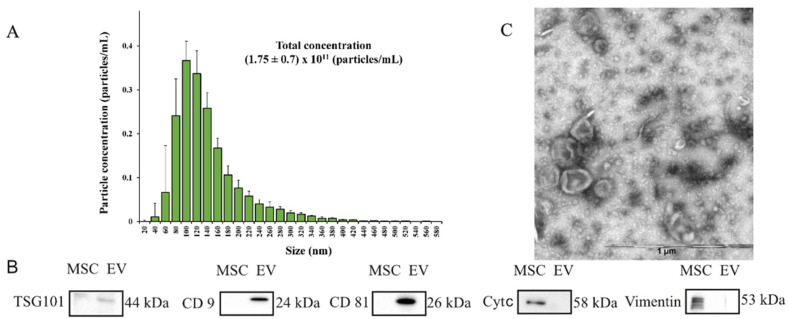
Characterization of EVs (produced by MSCs) in conditioned medium for 24 h of cultivation. (**A**) Particle size distribution of EV samples, obtained by differential centrifugation with a mean diameter of 113 ± 32 nm. N = 5; SEM = 0.23. (**B**) Evaluation of EVs markers (CD9, CD81, and TSG101) and non-EV markers (vimentin and cytochrome c) by Western blot analysis. (**C**) Transmission electron microscopy of EVs preparation.

The obtained EVs were characterized via NTA analysis, Western blotting to assess the content of EVs markers, and electron microscopy to evaluate the qualitative characteristics of the obtained samples (Figure 1).

The NTA analysis showed that the EVs were relatively homogeneous particles, with a size distribution of 20–420 nm (particles with diameters ranging from 80 to 140 nm represented the largest fractions) and an average particle diameter of 113 ± 32 nm. The total concentration of particles in the EV samples obtained from the conditioned MSC medium was (1.75 ± 0.7) × 10^11^ particles/mL (Figure 1A). Electrophoresis (SDS–PAGE) and Western blotting were applied to assess the content of EVs marker proteins, which detected characteristic markers such as CD9, CD81, and TSG101 tetraspanins in the EVs’ composition. However, the EVs’ lysate did not contain detectable amounts of vimentin or cytochrome c, which were detected in MSCs (Figure 1B). Transmission electron microscopy of the EVs preparation adhered to nitrocellulose carbon-coated stacks (Figure 1C) confirmed that most of the particles had a typical cup-shaped EV morphology, with the size ranging from 40 to 250 nm.

### 2.2. Effect of Extracellular Vesicles on the Atrophy of the Gastrocnemius Muscle in the Injured Limb

**Figure 2:**
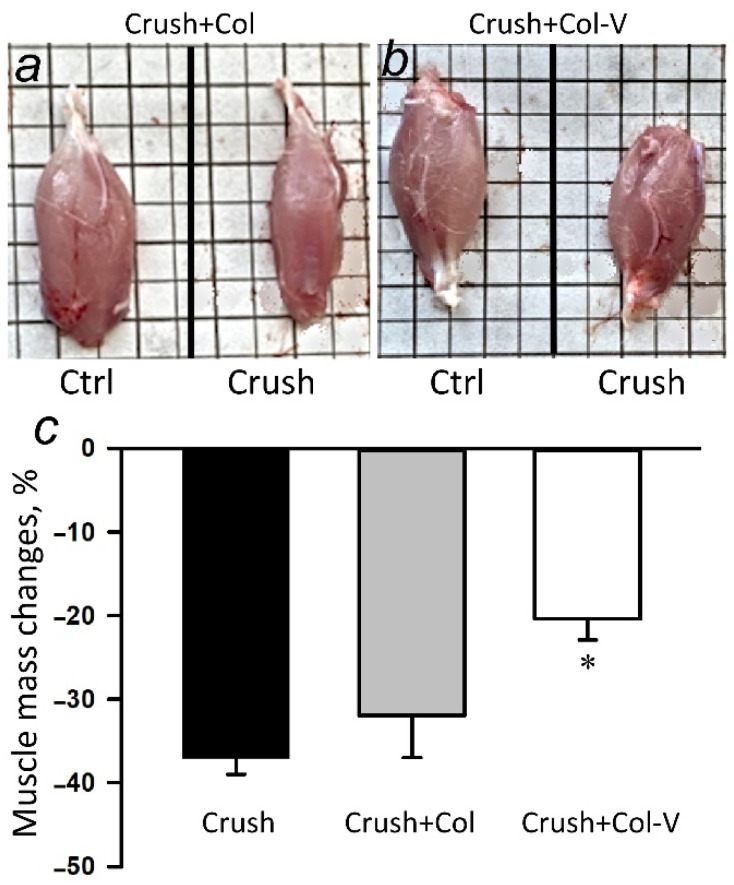
Weight loss of the gastrocnemius muscle of the injured limb relative to the weight of the muscle of the intact limb 30 days after sciatic nerve crush. (**a**) Left, view of the intact limb muscle (Ctrl); right, view of the muscle 30 days after the sciatic nerve crush (Crush). (**b**) Left, view of the intact limb muscle (Ctrl); right, view of the muscle 30 days after sciatic nerve crush and application of collagen–vesicle preparation 1 h after injury (Crush). (**c**) Change in gastrocnemius muscle mass 30 days after sciatic nerve crush (Crush), application of collagen gel (Crush+Col) and collagen–vesicle preparation (Crush+Col-V) in % relative to the contralateral muscle mass. M ± SEM; *n* = 8 (the number of animals in each experimental group); * *p* < 0.05 as compared to the group with crushing without treatment (Crush), One-Way ANOVA with Tukey’s post hoc test.

After the sciatic nerve crushing, atrophy of the gastrocnemius muscle, innervated by the injured nerve, occurs, and it is manifested by a significant decrease in muscle mass. The application of gel containing the vesicles to the injured area of the nerve reduced atrophy of the gastrocnemius muscle of the injured limb: the loss of muscle weight at day 30 was reduced by 19% (*p* ≤ 0.05) compared to the group with crushing (Figure 2).

**Figure 3:**
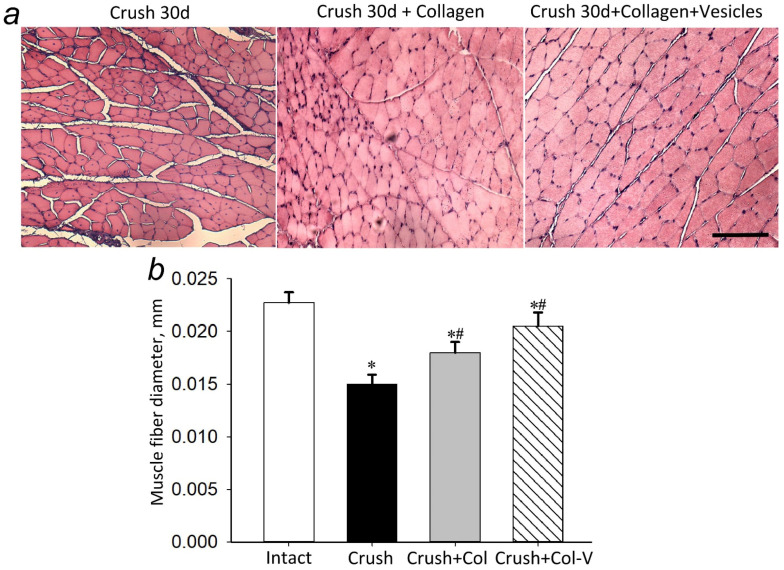
Changes in the diameter of muscle fibers of the gastrocnemius muscle of the injured limb 30 days after sciatic nerve crushing. (**a**) View of muscle fibers 30 days after sciatic nerve crushing (Crush 30 d), application of collagen gel (Crush 30 d+Collagen), and collagen preparation of vesicles (Crush 30 d+Collagen+Vesicles) one hour post-injury. Hematoxylin-and-eosin staining. Scale: 100 μm. (**b**) Changes in the diameter of muscle fibers of the gastrocnemius muscle 30 days after sciatic nerve crush (Crush), application of collagen gel (Crush+Col), and collagen preparation of vesicles (Crush+Col-V) one hour post-injury, in mm. M ± SEM; *n* = 8 (the number of animals in each experimental group); * *p* < 0.05 compared to the group with crushing without treatment (Crush); # *p* < 0.01 compared to the intact muscles (Crush+Col), One-Way ANOVA with Tukey’s post hoc test.

Another parameter of atrophy is a decrease in muscle fiber diameter. Our studies showed that a single application of collagen gel to the site of nerve damage affected the diameter of the muscle fiber; this parameter in the group with collagen treatment was 19.3% (*p* ≤ 0.05) higher than in the group with crushing alone. The application of vesicles as part of the collagen gel increased the diameter by 33.3% (*p* ≤ 0.05) relative to the group with crushing (Figure 3).

### 2.3. Functional Deficit of the Injured Limb

The functional deficit was assessed by using the extensor postural thrust test, where the paw pressure force on the scale surface is measured. At 30 days after the sciatic nerve crush, a 39% (*p* ≤ 0.05) increase in functional status was observed in the group with application of gel containing vesicles compared to the group without application. In 3, 7, 15, and 23 days, the effect of the application was not expressed (Table 1).

### 2.4. Assessment of the Number of Apoptotic Cells in Dorsal Root Ganglia and Sciatic Nerve Segment in Rats

**Figure 4:**
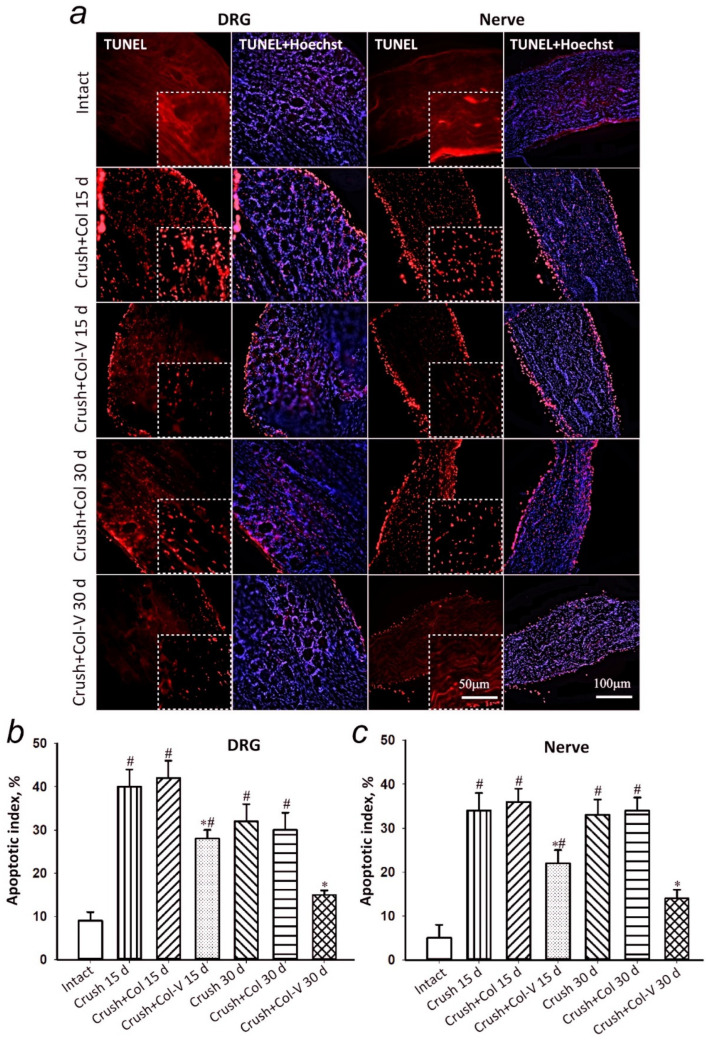
Apoptosis-level assessment in slices of the dorsal root ganglia and sciatic nerve. (**a**) Representative images of slices of the rat dorsal root ganglia (DRG) and a slice of the nerve (Nerve) at 0.5 to 1 cm from the crushing site, stained with TUNEL (red) and with both TUNEL and Hoechst 33342 (cell nuclei, blue) 15 and 30 days after the sciatic nerve crushing and application of gel-only application (Crush+Col) or gel–vesicle preparation (Crush+Col-V). Scale bar: 100 and 50 μm in the zoomed images. (**b**) Changes in apoptotic index in DRG. (**c**) Changes in apoptotic index in nerve slices. M ± SEM; *n* = 8 (mean apoptotic index in three DRG or nerve slices of eight animals of each experimental group); * *p* < 0.05 compared to the respective groups without vesicle treatment; # *p* < 0.05 compared to the intact nerve, One-Way ANOVA with Tukey’s post hoc test.

Damage to the sciatic nerve resulted in the development of apoptotic cell death both in the nerve itself and in the spinal ganglia which it extends from. It was detected by the appearance of a large number of TUNEL-positive cells in these structures 15 days after the injury (Figure 4). When the gel–vesicle preparation was applied to the crushed rat sciatic nerve, apoptosis dorsal root ganglia (DRG) was decreased by 33% (*p* < 0.05), and apoptosis of cells in the nerve adjacent to the injury site was decreased by 39% (*p* < 0.05) as compared to the group that did not receive the gel–vesicle preparation or gel-only application (Figure 4). In 30 days, the effect of the preparation was even more expressed, and this was probably due to the preparation’s effect on the mechanisms of natural regeneration. Thus, a 50% (*p* < 0.05) decrease in the number of apoptotic cells in DRG and a 59% (*p* < 0.05) decrease of that in the nerve segment (0.5–1 cm from the crushing site) were observed (Figure 4). There were no statistically significant differences between the group with the crushed nerve and the group that received the gel without vesicles after both 15 and 30 days after the injury.

### 2.5. Changes in the Levels of Regeneration-Associated Proteins

Nerve damage is characterized by changes in the expression of many genes, including the decrease in neurofilament (NF) proteins, as well as the disturbance in the distribution of cytoskeleton proteins. The immunohistochemical detection of NF-160 (medium-molecular-weight neurofilaments, 140–160 kDa) and NF-200 (high-molecular-weight neurofilaments, 195–200 kDa) is widely used to study regeneration of injured peripheral nerves [23,24]. Immunoreactivity to NF-200 was detected in 15% of all neurons in the DRG of intact rats. The number of the NF-200+ subpopulation of neurons is determined by the presence of connection to peripheral target cells and the ability to release neurotrophic factors predominantly from Schwann cells [25]. In this connection, an increase in NF-200 levels promotes neurite regeneration and maintains the phenotype of neurons that can contact the muscle spindle [24].

The growth-associated protein 43 (GAP-43), a neural protein localized in axons, can be expressed in all subpopulations of small and large DRG neurons. It appears to help neurons find their way and branch in the course of development and regeneration. Increasing GAP-43 is a marker of nerve regeneration or active axonal outgrowth after traumatic injury in vivo [26]. It was shown that the increase in GAP-43 expression in nerve damage correlates with the increase in tubulin synthesis, which correlates with the increase in the amount and rate of beta-III-tubulin delivery to axons of regenerating DRG neurons [27].

Eventually, regenerating axons in the peripheral nervous system can regain contact with their target, but many neurons die. Therefore, we analyzed the effect of collagen gel vesicle preparation (Col-V) on the level of nerve regeneration markers, namely 200 kDa neurofilament (NF) proteins, GAP-43, and βIII-tubulin, in rat dorsal root ganglion cells, at different times after sciatic nerve crushing.

**Figure 5:**
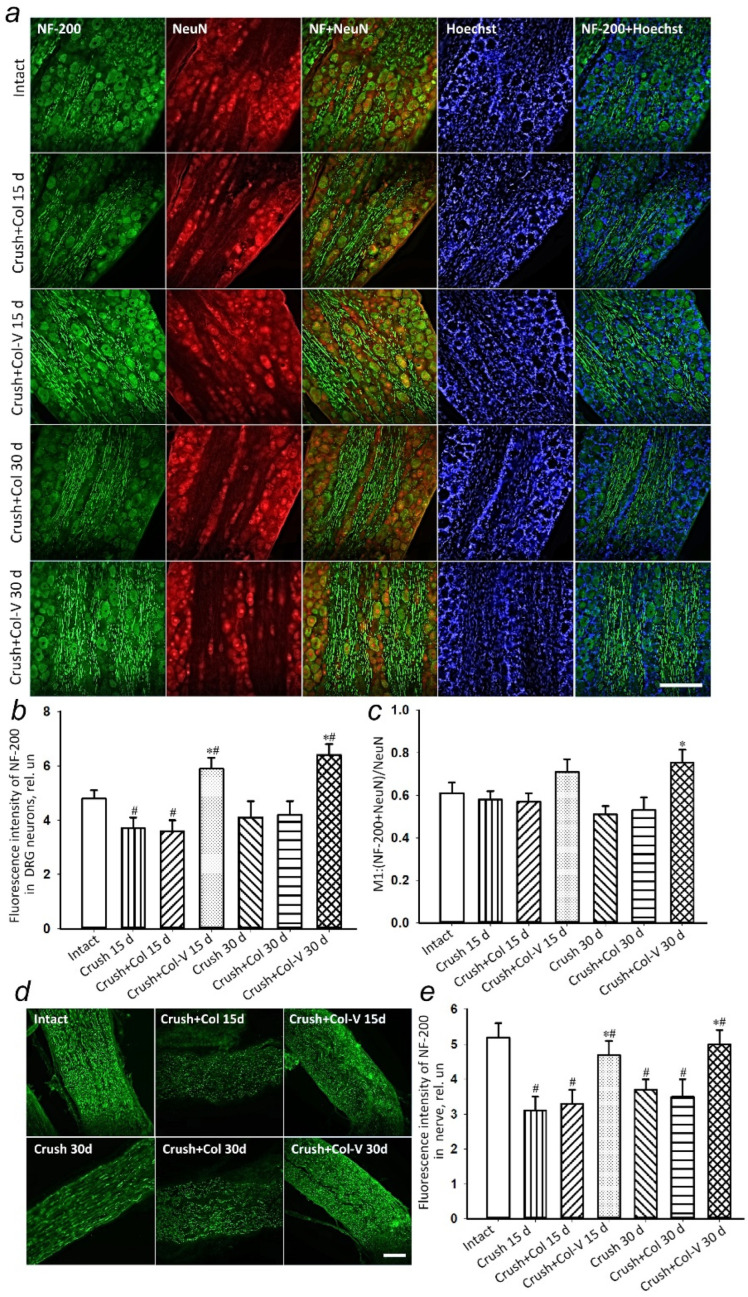
(**a**) Representative immunofluorescence images of NF-200 protein (green), combined fluorescence images of NF-200 and NeuN (red, neuronal marker), and combined fluorescence images of NF-200 and Hoechst (nuclear marker, blue) in rat dorsal root ganglia (DRG) 15 days (Crush and Crush+Col 15 d) and 30 days (Crush and Crush+Col 30 d) after injury and with applied only gel or vesicle gel preparation applied to the injured nerve (Crush +Col-V 15 d and 30 d, respectively). Scale bar: 50 μm. (**b**) Relative fluorescence intensity of NF-200 in DRG neurons at different times after crush, without and with the vesicle preparation applied. (**c**) Changes in Manders’ M1 colocalization coefficient of NF-200 protein and neuronal marker NeuN. (**d**) Immunofluorescence of NF-200 15 and 30 days in sciatic nerve after the injury: Int, intact nerve; Crush, crushing without gel application; Crush+Col, crushing with collagen gel application without vesicles; Crush+Col-V, crushing with the application of vesicle-containing gel. (**e**) Relative fluorescence intensity of NF-200 in the sciatic nerve; *n* = 7 (mean fluorescence intensity of NF-200 in three DRG slices (at least 100 neurons) or the nerve (general immunofluorescence) from seven animals of each experimental group). M ± SEM; * *p* < 0.05 compared with respective groups without vesicle treatment; # *p* < 0.05 compared to intact nerve, One-way ANOVA with Tukey’s post hoc test.

A statistically significant decrease in the level of NF-200 by 23% (*p* < 0.05) in the dorsal root ganglia was observed 15 days after the sciatic nerve crush and gel-only application (Crush+Col) compared to the group without damage (Figure 5a,b). There were no statistically significant differences between the group with the crushed nerve and the group that received the gel without vesicles after both 15 and 30 days after the injury.

The use of the vesicle gel (Col-V) preparation promoted a 64% (*p* < 0.05) increase in protein levels after 15 days and a 52% (*p* < 0.05) increase after 30 days compared to the groups that received the vesicle-free gel preparation. Moreover, the protein colocalization analysis with the neuronal marker NeuN indicated the growth of the subpopulation of neurons with NF200+ phenotype. After 30 days, there was a 54% (*p* < 0.05) increase in the colocalization coefficient of NF-200 with NeuN (Figure 5c).

The appearance of NF-200 (a marker of neurons forming A-fibers) is a critical event in axon stabilization, accompanying their maturation and myelination [23]. Immunohistochemical detection of NF-200 makes it possible to judge the proper formation of mature myelinated axons in the site of nerve regeneration. An examination of the nerve fragment at the distance of 0.5–1 cm from the damage site showed a 40% (*p* < 0.05) decrease in the level of NF-200 after 15 days in the group without gel application (Crush 15 d) and by 37% (*p* < 0.05) in the group with applied collagen gel without vesicles (Crush+Col) compared to intact nerves (Figure 5d,e). The low level of NF-200 in the sciatic nerve tissue persisted even 30 days after crushing (Figure 5d,e). The application of collagen gel containing vesicles increased the level of NF-200 by 42% (*p* < 0.05) after 15 days and by 43% (*p* < 0.05) after 30 days compared to the use of collagen gel without vesicles (Figure 5d,e).

**Figure 6:**
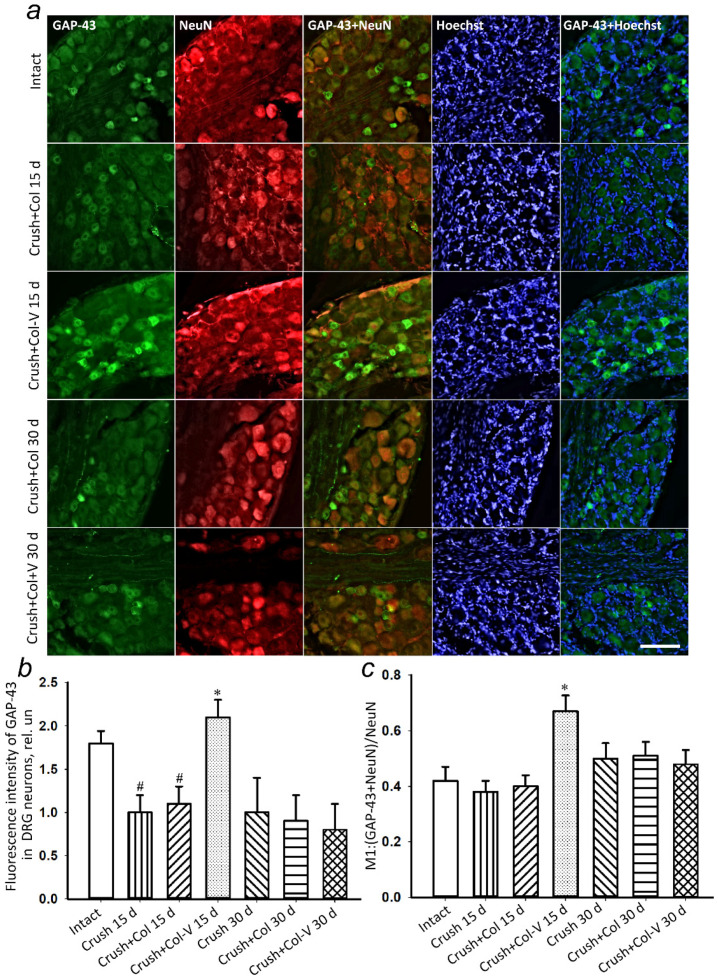
(**a**) Representative immunofluorescence images of GAP-43 protein (green), combined fluorescence images of GAP-43 with NeuN (red, neuronal marker), and GAP-43 with Hoechst (nuclear marker, blue) in rat DRG 15 days (Crush and Crush+Col 15 d) and 30 days (Crush and Crush+Col 30 d) after injury, without and with vesicle preparation applied to the injured nerve. Scale bar: 50 μm. (**b**) Relative fluorescence intensity of GAP-43 in DRG neurons at different times after crush, with and without vesicle preparation applied. (**c**) Changes in Manders’ colocalization coefficient M1 of GAP-43 protein with neuronal marker NeuN; *n* = 7 (mean fluorescence intensity of GAP-43 in three DRG slices (at least 100 neurons) or the nerve (general immunofluorescence) from seven animals of each experimental group). M ± SEM; * *p* < 0.05 compared with respective groups without vesicle treatment; # *p* < 0.05 compared to intact nerve, One-Way ANOVA with Tukey’s post hoc test.

The immunohistochemical staining for GAP-43 protein was used to detect regenerating axons. Fifteen days after the crushing of the sciatic nerve, there was a statistically significant increase in GAP-43 protein in the dorsal root ganglia of rats with a single application of the vesicle preparation by 96.3% (*p* ≤ 0.05) relative to the group without vesicle application (Figure 6a,b). The change in the level of GAP-43 colocalization with the neuronal marker (NeuN) has similar dynamics (Figure 6c), indicating an increase in GAP-43 expression, specifically in neurons.

In the course of the study, no changes were observed in the levels of GAP-43 protein in DRG 30 days after sciatic nerve injury and application of collagen gel containing vesicles compared to those of the groups without gel application and with application of gel containing no vesicles only (Figure 6).

The increase in GAP-43 expression in nerve damage was shown to correlate with an increase in tubulin synthesis [28]

**Figure 7:**
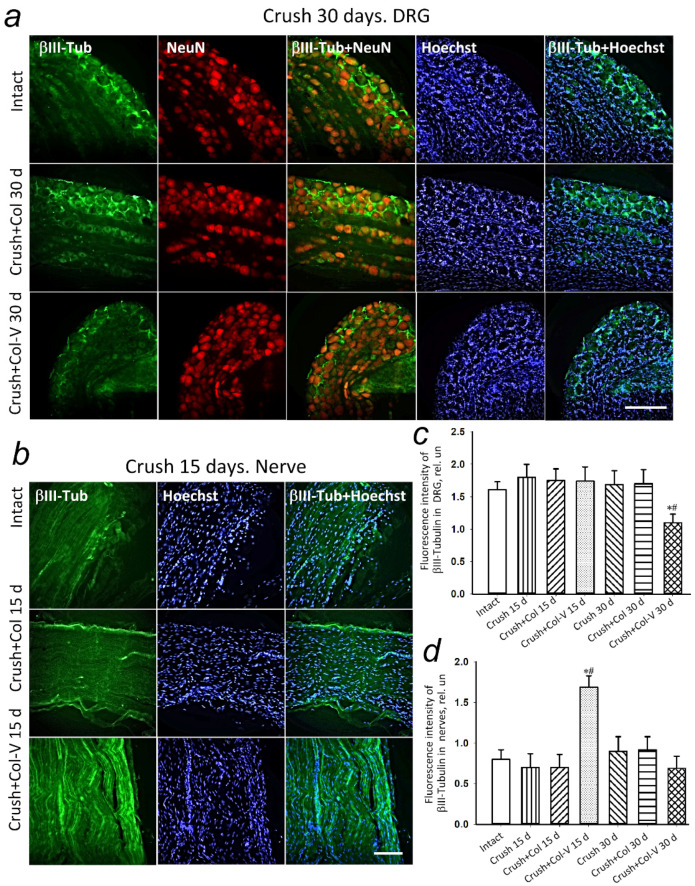
(**a**) Representative immunofluorescence images of βIII-tubulin protein (green) in dorsal root ganglia (DRG) of intact rats and 30 days after nerve crush (Crush), with gel-only application (Crush+Col), with vesicle treatment (Crush+Col-V), and combined fluorescence images of βIII-tubulin with NeuN (red, neuronal marker) and βIII-tubulin and Hoechst (nuclear marker, blue). Scale bar: 50 μm. (**b**) Representative immunofluorescence images of βIII-tubulin protein (green) in the sciatic nerve site (Nerve) of intact rats and 15 days after nerve crush (Crush), with only gel application (Crush+Col), with vesicle treatment (Crush+Col-V), and combined fluorescence images of βIII-tubulin with Hoechst (nuclear marker, blue). Scale bar: 50 μm. (**c**) Relative fluorescence intensity of βIII-tubulin in the dorsal root ganglia (DRG) of rats at different times after crushing, with and without vesicle treatment. (**d**) Relative fluorescence intensity of βIII-tubulin in the nerve (Nerve) at 0.5–1 cm from the injury site in rats at different times after crushing, with and without vesicle treatment; *n* = 7. M ± SEM; * *p* < 0.05 compared with respective group without vesicle treatment; # *p* < 0.05 compared to intact nerve, One-Way ANOVA with Tukey’s post hoc test.

The expression of βIII-tubulin in rat DRG 15 days after vesicle application to the crushed nerve site did not change compared to control groups (Figure 7a,b). The experiments showed a statistically significant decrease in the level of βIII-tubulin in rat DRG with a single vesicle gel application by 34.7% (*p* ≤ 0.05) relative to the group with the application of no-vesicle gel and by 32% (*p* ≤ 0.05) as compared to the group without application 30 days after sciatic nerve crush (Figure 7a,b). However, the level of βIII-tubulin at a distance of about 1 cm from the injury site increased by 53% (*p* ≤ 0.05) 15 days after nerve crushing and application of collagen vesicle preparation, as compared to the group that received collagen gel containing no vesicles (Figure 7a,c). Thirty days after nerve crushing, there were no significant differences in the levels of βIII-tubulin in the injured area of the sciatic nerve where the vesicle preparation was applied, as compared to the group without the application and with the gel containing no vesicles (Figure 7a,c).

## 3. Discussion

The effective functioning of EVs in recipient cells depends on their integration into the damaged peripheral nerve tissue. This study evaluated the efficacy of collagen gel implantation with integrated EVs when treating the consequences of sciatic nerve crush in the rat hindlimb.

Sciatic nerve injury has several negative aspects: death and dysfunction of nerve cells manifested in apoptosis, reduction of proteins responsible for axonal transport (motor proteins, adaptor proteins, and regulator proteins) [29] and as a result of the axonal transport itself, and reduced synthesis of regulatory proteins of regeneration and proliferation. All of this eventually leads to the decreased motor activity of the innervated muscle or its complete paralysis. In the course of performing the tests, we found that the atrophy of the injured limb in the group with transplanted EVs recovered by an average of 19% as compared to the group without treatment (Figure 2). At the same time, in the course of evaluation of functional indices of the muscle work, it was noticed that EVs promoted the reduction of the functional deficit by 39% in comparison with the group without therapeutic intervention after 30 days (Table 1).

In the literature, more and more data on the neuroprotective role of the EVs produced by MSCs appear, and, similar to the cells themselves, EVs apparently act in several directions: immunoregulation, which promotes inhibition of inflammatory microenvironment and triggers repair processes; remodeling of the damaged area; and the stimulation of neuronal progenitor precursor cells to oligodendrocyte differentiation [30,31,32]. In the course of the recovery of damaged peripheral nerve in this work, our attention was focused specifically on the regeneration. First, the anti-apoptotic protective role of EVs was confirmed experimentally, as soon as the number of apoptotic cells in the dorsal root ganglia actually reached the control level by 30 days after the start of therapy (Figure 4). It is known that about a third of the neurons in the DRG and a significant part of the glial cells die within weeks or months after sciatic nerve injury [33,34,35]. Glial cell apoptosis is observed as early as 24 h after sciatic nerve transection and intensifies on the seventh day, when apoptosis of some neurons just begins. Neuronal death is modulated by peripheral nerve repair and its time after axotomy.

Probably, the restoration of the pool of necessary neurons and, respectively, their neuronal extensions maintaining the necessary level of innervation is one of the explanations of the restoration of the neuromuscular fiber diameter by 33.3% as compared to the group without therapy (Figure 3). In turn, the neuroprotective effect can be associated both with the initiation of neuronal progenitors’ differentiation and with the increase in the synthesis of regeneration initiator proteins, such as NF-200, GAP-43, and βIII-tubulin. It is known that nerve damage primarily disturbs the synthesis of neurofilaments and changes the expression profile of cytoskeleton proteins necessary for maintaining the growth of neuronal extensions, primarily axons. The studies showed that, after 30 days, EVs increased the synthesis of NF-200 in neurons of DRG by more than 60% compared to the group without treatment (gel without vesicles; see Figure 5). Axonal remodeling is a prerequisite for successful restoration of functional activity of the neuromuscular system. For this purpose, we evaluated the level of synthesis of a specific membrane-bound phosphoprotein contained in the growing axons and being the main component of growth cones, GAP-43. Axonal growth is implemented with the involvement of several specific neuronal proteins. Protein gene product 9.5 (PGP 9.5) and GAP-43 protein are most commonly used as markers for immunohistochemical detection of regenerating axons in experimental studies. GAP-43 is a membrane-bound phosphoprotein found in growing axons and is a major component of growth cones. Similar to other axonal proteins, it is synthesized in perikaryons and transported to axons. Its expression increases in nerve cells during regeneration [36]. The obtained results have shown that, after 15 days, the level of GAP-43 synthesis increased by almost 97% compared to the group without EVs treatment, indicating a high intensity of axonal growth, but by day 30, no statistical changes were found in any of the three groups. The formation of mature myelinated axons at the site of nerve regeneration is also indicated by the growth of NF-200 level as early as 15 days after the application of vesicle preparation (Figure 5d,e). This may indicate, perhaps, the depletion of the EVs’ therapeutic potential in the context of axonal growth. It is also indirectly confirmed by the data on βIII-tubulin, a protein that is found in higher amounts specifically in the neuronal outgrowths [37]. βIII-tubulin has been described in both central and peripheral nervous system cells. It is a structural protein of neurotubules, and one of its functions in neurons is to implement axonal transport. It was found that the highest concentration of beta-III-tubulin was observed in neuronal outgrowths, but not in perikaryons [38]. A high content of beta-III-tubulin was observed in peripheral nerve conductors. Immunohistochemical detection of these proteins in axons of peripheral nerves allows studying their regeneration on different models, for example, on models using scaffolds, special carriers of cultured cells [39]. A slight decrease in its synthesis in the ganglion tissues was detected on day 15 after the start of treatment, and this may indirectly indicate a decrease in axonal growth and decelerating regeneration, which slows down even more noticeably by day 30 from the start of therapy (Figure 6 and Figure 7). However, the effect of the preparation on the muscle function recovery is evident only by day 30.

It should be noted that we used collagen gel as the delivery method for extracellular vesicles, and the gel is not biologically neutral and may have its own therapeutic effect. Indeed, according to some of the estimated parameters, we observed a moderate pronounced effect of collagen gel. This fact could be explained by several mechanisms of therapeutic action. A collagen gel can act as a barrier to prevent leukocytes from entering the injury site [40]. We also do not rule out the presence of a second mechanism associated with collagen degradation products, primarily peptides. It is known that matrix metalloproteinases can cleave collagen and form biologically active peptides that may have neuroprotective effects, including the promotion of neurite outgrowth [41] or neural progenitor cell proliferation [42]. However, the neuroprotective effect of vesicles was more significantly pronounced than only in the group treated with collagen gel.

Thus, the obtained data clearly indicate the effectiveness of EVs in collagen gel for peripheral nerve repair in the early stages (15–30 days) of pathology development. However, at the same time, it was revealed that the effect is not long-term, and its prolongation may require repeated procedure, increasing the number of EVs injected into the gel, or using a nerve conduit [43]. In addition, this work did not assess the inflammatory component accompanying the lesion in any way; therefore, we were unable to clearly judge the state of the microenvironment at 30 days after the start of the treatment; perhaps, this information would have brought clarity to the deceleration of regenerative effects. It should also be taken into account that the action of biologically active factors contained in EVs, be they proteins, lipids, or miRNA, is limited in time, and further evaluation of the secretome via proteomic and miRNA analysis in order to detect neuroprotection-significant molecules is necessary. This knowledge will allow the creation of modified EVs loaded with molecules important for neuroprotection, which may improve the therapeutic effect, and allow us to proceed to clinical trials in this context.

## 4. Materials and Methods

### 4.1. Primary Culture of MSCs

Human postpartum placenta samples were obtained from healthy women 22 to 26 years old (*n* = 5) who delivered healthy full-term infants by cesarean section at the V.I. Kulakov National Medical Research Center for Obstetrics, Gynecology, and Perinatology. These women had no history of infectious diseases or pregnancy complications and were confirmed to be negative for hepatitis B virus (HBV), human immunodeficiency virus (HIV), and syphilis.

The internal part (approximately 1 cm^3^) of central placenta lobules was washed in phosphate-buffered saline (PBS) (Paneco, Moscow, Russia) several times, cut into small fragments, and enzymatically digested with 100 U/mL collagenase type I (Gibco) in serum-free Dulbecco’s Modified Eagle Medium (DMEM) (Paneco, Moscow, Russia). Cell suspensions from collagen digests were collected by centrifugation for 5 min, at 300× *g*, and then washed in DMEM and pelleted again by 5 min centrifugation, at 300× *g*. The cells were suspended in DMEM/F12 (Paneco, Moscow, Russia) (1:1) containing 7% fetal bovine serum (FBS) (Biosera, Nuaille, France) supplemented with penicillin (100 IU/mL), streptomycin (100 μg/mL) (Gibco, Grand Island, NY, USA), and 2 mM L-glutamine (Paneco, Moscow, Russia). Prior to use, the culture medium was centrifuged at 108,000× *g* for 1.5 h at 4 °C by an Avanti JXN-30 high-speed centrifuge (Beckman Coulter Inc., Fullerton, CA, USA) to avoid possible contamination with EVs that originated from FBS. Then the supernatant was harvested, filtered by using a bottle-top vacuum filter system with a pore size of 0.22 μm (Falcon, Corning, NY, USA), and used for further experiments as vesicle-free culture medium. Initially, cells were plated into a single 75 cm^2^ tissue culture flask (Gibco Life Technologies, Baltimore, MD, USA). Cells were incubated in a humidified atmosphere with 5% CO_2_ at 37 °C. The incubation medium was refreshed every 3 to 4 days to remove nonadherent cells. Cell growth and morphology were monitored daily, using an inverted microscope. MSCs at the third-to-fourth passage were used for harvesting extracellular vesicles in three-layer flasks (Nunc, Thermo Fisher Scientific, Dreieich, Germany).

### 4.2. Isolation of Extracellular Vesicles by Differential Centrifugation

EVs were isolated from MSC-cultured media, following the guideline recommended by ISEV (International Society for Extracellular Vesicles), called MISEV2018 (Minimal Information for Studies of Extracellular Vesicles 2018) [44]. Differential centrifugation was used for the isolation of EVs, as described previously [45]. Briefly, supernatants were collected from conditioned medium of MSCs culture at 80–90% confluence (~25 × 106 cells) 24 h after medium refreshment. Conditioned medium (50 mL) from confluent cultures was collected and processed by using serial centrifugations to remove cells and debris (400× *g* for 10 min, followed by 10,000× *g* at 4 °C for 30 min). Supernatant was used for EVs isolation by ultracentrifugation at 108,000× *g* for 1.5 h at 4 °C, with further pellet washing with PBS, followed by another spin at 108,000× *g* for 1.5 h to minimize protein contamination. The final EVs pellet was resuspended in 30 μL of PBS for experiments in vitro and in vivo and 1 mL for nanoparticle tracking analysis (NTA). Vesicle samples were stored at −80 °C.

### 4.3. Analysis of the Size of Extracellular Vesicles

The size distribution of vesicles was measured by nanoparticle tracking analysis (NTA), using a NanoSight LM10 (Malvern Panalytical, London, UK) equipped with a blue laser (405 nm, 60 mW) and CMOS camera (Hamamatsu Photonics K.K., Hamamatsu City, Japan). An EVs pellet resuspended in 1 mL PBS was diluted 10,000 times by serial dilution with PBS to reach the optimal concentration for instrument linearity (20–30 particles/frame), and readings were performed in triplicates of 30 s at 30 frames per second. For the analysis of concentration and size distribution, 12 exposures of 60 s each were recorded with 15–22 particles per image. Data were analyzed with NTA 2.3 software (Malvern Panalytical, Malvern, UK), using the following settings: calibration, 166 nm/pixel; blur, auto; detection threshold, 8, minimum track length, auto; minimum expected particle size, 30 nm; temperature, 24.7 °C; and viscosity, 0.90 cP. Calibration was performed by using Nanosight size transfer standards (100, 200, and 400 nm) and Nanosight fluorescence standards (Malvern Panalytical, Malvern, UK).

### 4.4. Animals

The sciatic nerve crush injury was performed on adult male Wistar rats (200–250 g), which were kept in cages in groups of 4 or 5 animals, with free access to food and water, in standard vivarium conditions: temperature, 22–25 °C; light conditions 12 h light/12 h dark; and air exchange rate, 18 changes per hour. Rats were acclimatized in the environment for one week prior to the experiment.

### 4.5. Rat Sciatic Nerve Crushing Model

The rats were anesthetized via intramuscular injection of 1.7 mL of a 2:1 mixture of Xyla and Telazol: 2% xylazine hydrochloride solution, (Interchemie Werken “de Adelaar” BV, Venray, The Netherlands); and telazol, a mixture of tiletamine hydrochloride and zolazepam hydrochloride (Zoetis, Parsippany-Troy Hills, NJ, USA).

**Figure 8:**
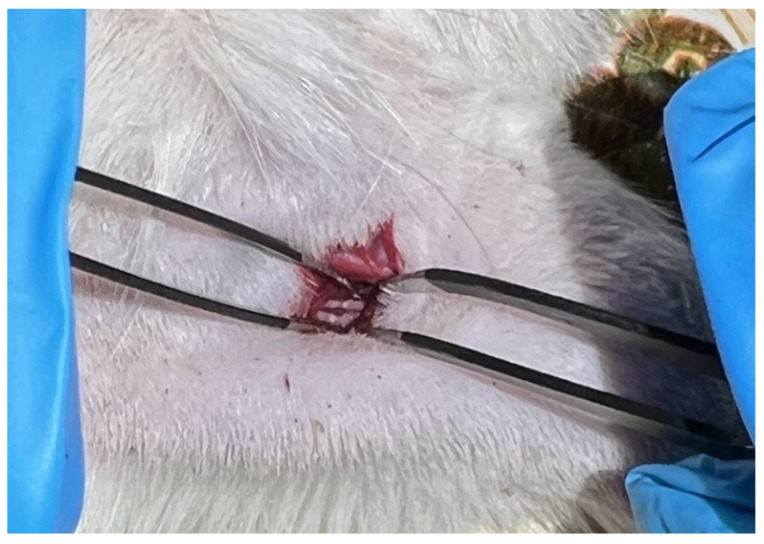
The photo shows the result of sciatic nerve crushing, dark areas at the site of axon rupture.

Before the surgery, the lateral surface of the sacrum and femur of the rat’s right hindlimb was shaved. The sciatic nerve on the left side remained intact and served as a control. Then the animal was placed ventral-side down on the working surface equipped with mounts for fixation of the limbs. It is necessary to fix the rat’s hindlimbs in a sufficiently stretched position, but without overzealousness and causing harm to the animal. This position reduces the thickness of the muscle overlying the nerve and straightens the nerve itself, thus making it easier to find. Next, local disinfection of the shaved skin area was performed. Then a 1.5 cm skin incision was made with forceps and scissors. Then the scissors were used to press vertically on the exposed muscle so that they could pass into the cavity, where the nerve lies. The scissors were placed 1 cm distal to the femur (trochanter major) and 0.5–1 cm orthogonal in the caudal direction. At this time, cutting movements should be avoided, and an opening in the muscle should be just due to the pressure on the scissors. After the scissors fell into the cavity, they were gently opened, increasing the visibility area. Then a 3 mm–long segment of the sciatic nerve was crushed by maximal squeezing of the nerve with non-serrated hemostatic forceps (model 65-H) for 20 s, without displacing it. The nerve in the place of impact would become transparent, as the axons, but not the nerve sheath, would form a rupture in the place of injury (axonotmesis) (Figure 8).

Collagen gel, with and without vesicles, was applied to the nerve crushed in this manner. After the surgery, the skin was sutured by using a simple knot stitch applied every 4–5 mm. Then, after 15 and 30 days in the group with crushing trauma, animals were decapitated under anesthesia, followed by collection of material, viz 4 and 5 spinal ganglia, the injured area of the sciatic nerve at a distance of 0.5–1 cm from the injury site, and the area of the calf muscle.

### 4.6. Preparing Collagen-Gel-Based Vesicle Substance

To prepare collagen-gel-based vesicle substance, we dissolved 1.5 g of I and III type collagen (hydrolyzed bovine collagen Doctor’s Best Pure Collagen Types 1 and 3 Powder) in 1 mL of water for injection, and we took from there 50 µL per each rat and added 3 µL of vesicle solution. The collagen-gel-based substance was applied in one hour after nerve crushing to the damaged nerve, around the nerve injury site.

### 4.7. Estimation of Gastrocnemius Muscle Mass Change in Damaged Limb in 30 Days after Sciatic Nerve Crush

As a consequence of sciatic nerve damage, a gradual decrease in the muscular mass of the damaged limb takes place, including that of the gastrocnemius muscle. We estimated the percent of gastrocnemius mass change in the group with nerve crush, in the group where only gel was applied on the damaged nerve site, and in the group where the vesicle-containing gel was applied. The obtained data made it possible to evaluate to what extent this intervention helps to improve regeneration and reduce functional deficit after the injury.

### 4.8. Extensor Postural Thrust Test

The physiological extensor postural thrust test was performed on day 7, 15, 23, and 30 after rat sciatic nerve crushing and administration of gel-based vesicle substance. The test was developed to study recovery of nerve function after sciatic nerve transection and is based on measuring strength, applied to digital scale, expressed in grams [46]. The instinctive extension of a limb, when the rat is brought near the scale surface, occurs via the work of soleus and gastrocnemius muscles. The rat’s body is wrapped in a towel, only leaving alternately healthy and experimental limbs, while holding the animal vertically. In the course of descending to the platform, it extends the hindlimb, expecting the contact with the scale surface. The force, applied by the injured hindlimb to the scale, is measured. The force, applied by the healthy hindlimb, serves as a control. The rate of functional deficit is calculated by the formula [(experimental value–control value)/control value] × 100%.

### 4.9. Preparing Muscle Tissues for Histological Study

For the histological study, the tissue fragments (2 × 2 × 0.5 cm), 15 or 30 days after nerve crushing, were placed in 10% neutral buffered formalin solution and were fixed in no less than 24 h. The histological processing was performed in an automatic tissue processor (Leica TP1020, Leica Biosystems, Nußloch, Germany). After the processing, tissue samples were embedded in paraffin by using an embedding station (Leica EG1150H, Leica Biosystems, Nußloch, Germany). From paraffin blocks with the tissue samples, 3–5 µm serial slices were made and placed on microscope slides with antiadhesion coating (Menzel-Glaser). The obtained slices of tissue samples were stained with hematoxylin and eosin by classic protocol and examined under a Nikon Eclipse FN1 microscope (Nikon, Tokyo, Japan). The estimation of the axon number and muscle fiber diameter was performed by using the ImageJ software (NIH, Bethesda, MD, USA, https://imagej.nih.gov/ij/index.html (accessed on 27 June 2022)).

### 4.10. Estimation of Apoptotic Cell Number

The imaging of apoptotic cells was performed by the TUNEL method (TdT-mediated dUTP-X nick end labeling), marking DNA strand breaks by using the “In Situ Cell Death Detection Kit, TMR red” (#12156792910, Roche, Basel, Switzerland). For that, the slices were treated with the kit reagents according to the manufacturer’s recommendations, with the addition of Hoechst 33342 in a final concentration 10 µg/mL, and incubated for one hour at 37 °C. The apoptotic index (AI) was calculated by TUNEL-positive cell in the whole area of a sample at ×20 magnification by the formula AI = [TUNEL-positive cell number/total cell number (Hoechst 33342-stained)] × 100%. The vesicle-containing collagen-based gel substance was applied on the site of sciatic nerve crush damage. Spinal ganglia and sections of nerve were taken in 15 or 30 days after the injury.

### 4.11. Immunofluorescence Microscopy of Rat Spinal Ganglia and Damaged Nerve Fragments

Slices of rat DRG, 40 µm in width, after washing from sucrose in PBS, were incubated for one hour with 5% BSA and 0.3% Triton X-100 to block non-specific binding sites. Then the slices were incubated with primary anti-rabbit antibodies Anti-Neurofilament 200 (N4142), Anti-GAP-43 (SAB4300525), and Anti-βIII-Tubulin (T2200) at a dilution of 1:500, and with anti-mouse antibodies for neurospecific nuclear protein Anti-NeuN (MAB377), at a dilution of 1:1000, overnight, at 4 °C. After three-time washing in PBS, the slices were incubated with secondary antibodies anti-rabbit IgG (H+L), CF™ 488A (SAB4600045; 1:1000), and anti-mouse IgG1 (γ1), CF™555 (SAB4600302; 1:1000). All antibodies were purchased from Sigma-Aldrich. Cell nuclei were imaged with Hoechst 33342 (40 µM; 10 min). The slices were place in 60% glycerol and examined on the Olympus BX-51 fluorescence microscope (Olympus, Tokyo, Japan), with digital camera OrcaFlash 4.0 V3 (Hamamatsu, Japan), or in the Eclipse FN1 fluorescence microscope (Nikon, Japan). Data from measurements were normalized to background fluorescence intensity I_norm_= (I_mean_−I_back_)/I_back_, where I_mean_ is mean intensity in selected area, and I_back_ is mean background fluorescence. NF-200 and GAP-43 fluorescence intensity in DRG sections were assessed only in neurons, at least 100 cells for each animal. For βIII-Tubulin, the total fluorescence intensity in DRG and nerve sections was assessed. For each animal, three DRG slices (with 30–40 detectable neurons in each) and one nerve slice were examined, and the values obtained were averaged.

We also calculated Manders’ Coefficient M1 to estimate the colocalization of neurofilament 200 and β-Actin with neuron marker NeuN. Images where processed by using ImageJ software with “JACoP” plugin [47].

### 4.12. Data Processing and Statistical Analysis

The normalization of data to calculate the relative fluorescence intensity was performed in Microsoft Excel 365, using the abovementioned formulae. All values are given as the mean (M) ± standard error (SEM). For the results, presented in Figure 2, Figure 3, Figure 4, Figure 5, Figure 6 and Figure 7 and Table 1, the significance of inter-group differences was estimated by using One-Way ANOVA, with Tukey’s post hoc test. The statistical analysis and plotting were performed by using SigmaPlot v12.5 software (Systat Software, San Jose, CA, USA).

## Figures and Tables

**Table 1:** Functional deficit of the injured limb relative to the intact limb in % after sciatic nerve crushing.

Groups	3 d	7 d	15 d	30 d
Crush	−100 ± 2	−100 ± 2	−87 ± 8	−66 ± 12
Crush+Col	−100 ± 3	−100 ± 2	−100 ± 3	−50 ± 13
Crush+Col-V	−100 ± 1	−100 ± 2	−81 ± 10	−40 ± 12 *

Crush, sciatic nerve crush; Crush+Col, sciatic nerve crush with application of collagen gel to the damaged area; Crush+Col-V, sciatic nerve crush with application of gel containing vesicles to the damaged area; *n* = 8 (the number of animals in each experimental group); * *p* ≤ 0.05 compared to the Crush group, One-Way ANOVA with Tukey’s post hoc test.

## Data Availability

The raw data supporting the conclusions of this article will be made available by the authors, without undue reservation.

## References

[1] Sayad Fathi S., Zaminy A. (2017). Stem cell therapy for nerve injury. World J. Stem Cells.

[2] Palomo R., Sánchez R. (2020). Physiotherapy applied to the upper extremity in 0 to 10-year-old children with obstetric brachial palsy: A systematic review. Rev. Neurol..

[3] Van der Looven R., Le Roy L., Tanghe E., Samijn B., Roets E., Pauwels N., Deschepper E., De Muynck M., Vingerhoets G., Van den Broeck C. (2020). Risk factors for neonatal brachial plexus palsy: A systematic review and meta-analysis. Dev. Med. Child Neurol..

[4] Barsaoui M., Safi H., Said W., Nessib M.N. (2017). Nerve surgery in obstetric brachial plexus palsy, report of 68 cases. Tunis. Med..

[5] Moucharafieh R.C., Badra M.I., Boulos K.A., Mansour J.I., Daher J.C., Wardani H.M., Nour H.G.A., El Sayde E.G., Nehme A.H. (2020). Nerve transfers in the upper extremity: A review. Injury.

[6] Hsueh Y.-H., Tu Y.-K. (2020). Surgical reconstructions for adult brachial plexus injuries. Part I: Treatments for combined C5 and C6 injuries, with or without C7 injuries. Injury.

[7] Song L., Guo Q., Guo J., Xu X., Xu K., Li Y., Yang T., Gu X., Cao R., Cui S. (2022). Brachial plexus bridging with specific extracellular matrix-modified chitosan/silk scaffold: A new expand of tissue engineered nerve graft. J. Neural Eng..

[8] Gu X., Ding F., Williams D.F. (2014). Neural tissue engineering options for peripheral nerve regeneration. Biomaterials.

[9] Schlosshauer B., Dreesmann L., Schaller H.-E., Sinis N. (2006). Synthetic nerve guide implants in humans: A comprehensive survey. Neurosurgery.

[10] Deumens R., Bozkurt A., Meek M.F., Marcus M.A.E., Joosten E.A.J., Weis J., Brook G.A. (2010). Repairing injured peripheral nerves: Bridging the gap. Prog. Neurobiol..

[11] Burdick J.A., Mauck R.L., Gorman J.H., Gorman R.C. (2013). Acellular biomaterials: An evolving alternative to cell-based therapies. Sci. Transl. Med..

[12] Sullivan R., Dailey T., Duncan K., Abel N., Borlongan C. (2016). V Peripheral Nerve Injury: Stem Cell Therapy and Peripheral Nerve Transfer. Int. J. Mol. Sci..

[13] Rodríguez Sánchez D.N., de Lima Resende L.A., Boff Araujo Pinto G., de Carvalho Bovolato A.L., Possebon F.S., Deffune E., Amorim R.M. (2019). Canine Adipose-Derived Mesenchymal Stromal Cells Enhance Neuroregeneration in a Rat Model of Sciatic Nerve Crush Injury. Cell Transplant..

[14] Lopatina T., Bruno S., Tetta C., Kalinina N., Porta M., Camussi G. (2014). Platelet-derived growth factor regulates the secretion of extracellular vesicles by adipose mesenchymal stem cells and enhances their angiogenic potential. Cell Commun. Signal..

[15] Zhang Z.G., Chopp M. (2016). Exosomes in stroke pathogenesis and therapy. J. Clin. Investig..

[16] Harrell C.R., Jovicic N., Djonov V., Arsenijevic N., Volarevic V. (2019). Mesenchymal Stem Cell-Derived Exosomes and Other Extracellular Vesicles as New Remedies in the Therapy of Inflammatory Diseases. Cells.

[17] Raposo G., Stoorvogel W. (2013). Extracellular vesicles: Exosomes, microvesicles, and friends. J. Cell Biol..

[18] Blanc L., Vidal M. (2018). New insights into the function of Rab GTPases in the context of exosomal secretion. Small GTPases.

[19] Boltze J., Arnold A., Walczak P., Jolkkonen J., Cui L., Wagner D.-C. (2015). The Dark Side of the Force—Constraints and Complications of Cell Therapies for Stroke. Front. Neurol..

[20] Yu T., Xu Y., Ahmad M.A., Javed R., Hagiwara H., Tian X. (2021). Exosomes as a Promising Therapeutic Strategy for Peripheral Nerve Injury. Curr. Neuropharmacol..

[21] Tsuruta T., Sakai K., Watanabe J., Katagiri W., Hibi H. (2018). Dental pulp-derived stem cell conditioned medium to regenerate peripheral nerves in a novel animal model of dysphagia. PLoS ONE.

[22] Dong R., Liu Y., Yang Y., Wang H., Xu Y., Zhang Z. (2019). MSC-Derived Exosomes-Based Therapy for Peripheral Nerve Injury: A Novel Therapeutic Strategy. Biomed Res. Int..

[23] Guseva D., Chelyshev Y. (2006). The Plasticity of the DRG Neurons Belonging to Different Subpopulations After Dorsal Rhizotomy. Cell. Mol. Neurobiol..

[24] Zhang W., Li Z. (2013). The Effects of Target Skeletal Muscle Cells on Dorsal Root Ganglion Neuronal Outgrowth and Migration In Vitro. PLoS ONE.

[25] Lekan H.A., Chung K., Yoon Y.W., Chung J.M., Coggeshall R.E. (1997). Loss of dorsal root ganglion cells concomitant with dorsal root axon sprouting following segmental nerve lesions. Neuroscience.

[26] Murata R., Ohtori S., Ochiai N., Takahashi N., Saisu T., Moriya H., Takahashi K., Wada Y. (2006). Extracorporeal shockwaves induce the expression of ATF3 and GAP-43 in rat dorsal root ganglion neurons. Auton. Neurosci..

[27] Moskowitz P., Oblinger M. (1995). Sensory neurons selectively upregulate synthesis and transport of the beta III-tubulin protein during axonal regeneration. J. Neurosci..

[28] Fernandes K.J., Fan D.P., Tsui B.J., Cassar S.L., Tetzlaff W. (1999). Influence of the axotomy to cell body distance in rat rubrospinal and spinal motoneurons: Differential regulation of GAP-43, tubulins, and neurofilament-M. J. Comp. Neurol..

[29] Maday S., Twelvetrees A.E., Moughamian A.J., Holzbaur E.L.F. (2014). Axonal transport: Cargo-specific mechanisms of motility and regulation. Neuron.

[30] Cui Y., Liu C., Huang L., Chen J., Xu N. (2021). Protective effects of intravitreal administration of mesenchymal stem cell-derived exosomes in an experimental model of optic nerve injury. Exp. Cell Res..

[31] Riazifar M., Mohammadi M.R., Pone E.J., Yeri A., Lässer C., Segaliny A.I., McIntyre L.L., Shelke G.V., Hutchins E., Hamamoto A. (2019). Stem Cell-Derived Exosomes as Nanotherapeutics for Autoimmune and Neurodegenerative Disorders. ACS Nano.

[32] Mead B., Tomarev S. (2017). Bone Marrow-Derived Mesenchymal Stem Cells-Derived Exosomes Promote Survival of Retinal Ganglion Cells Through miRNA-Dependent Mechanisms. Stem Cells Transl. Med..

[33] McKay Hart A., Brannstrom T., Wiberg M., Terenghi G. (2002). Primary sensory neurons and satellite cells after peripheral axotomy in the adult rat. Exp. Brain Res..

[34] Vigneswara V., Berry M., Logan A., Ahmed Z. (2013). Caspase-2 Is Upregulated after Sciatic Nerve Transection and Its Inhibition Protects Dorsal Root Ganglion Neurons from Apoptosis after Serum Withdrawal. PLoS ONE.

[35] Dzreyan V., Rodkin S., Nikul V., Pitinova M., Uzdensky A. (2021). The Expression of E2F1, p53, and Caspase 3 in the Rat Dorsal Root Ganglia After Sciatic Nerve Transection. J. Mol. Neurosci..

[36] Sommervaille T., Reynolds M.L., Woolf C.J. (1991). Time-dependent differences in the increase in GAP-43 expression in dorsal root ganglion cells after peripheral axotomy. Neuroscience.

[37] McKerracher L., Essagian C., Aguayo A. (1993). Marked increase in beta-tubulin mRNA expression during regeneration of axotomized retinal ganglion cells in adult mammals. J. Neurosci..

[38] Braun H., Schäfer K., Höllt V. (2002). BetaIII tubulin-expressing neurons reveal enhanced neurogenesis in hippocampal and cortical structures after a contusion trauma in rats. J. Neurotrauma.

[39] Chung T.-W., Yang M.-C., Tseng C.-C., Sheu S.-H., Wang S.-S., Huang Y.-Y., Chen S.-D. (2011). Promoting regeneration of peripheral nerves in-vivo using new PCL-NGF/Tirofiban nerve conduits. Biomaterials.

[40] Burton V.J., Butler L.M., McGettrick H.M., Stone P.C., Jeffery H.C., Savage C.O., Rainger G.E., Nash G.B. (2011). Delay of migrating leukocytes by the basement membrane deposited by endothelial cells in long-term culture. Exp. Cell Res..

[41] McGrady N.R., Pasini S., Baratta R.O., Del Buono B.J., Schlumpf E., Calkins D.J. (2021). Restoring the Extracellular Matrix: A Neuroprotective Role for Collagen Mimetic Peptides in Experimental Glaucoma. Front. Pharmacol..

[42] Nogimura D., Mizushige T., Taga Y., Nagai A., Shoji S., Azuma N., Kusubata M., Adachi S., Yoshizawa F., Kabuyama Y. (2020). Prolyl-hydroxyproline, a collagen-derived dipeptide, enhances hippocampal cell proliferation, which leads to antidepressant-like effects in mice. FASEB J..

[43] Grinsell D., Keating C.P. (2014). Peripheral nerve reconstruction after injury: A review of clinical and experimental therapies. Biomed Res. Int..

[44] Théry C., Witwer K.W., Aikawa E., Alcaraz M.J., Anderson J.D., Andriantsitohaina R., Antoniou A., Arab T., Archer F., Atkin-Smith G.K. (2018). Minimal information for studies of extracellular vesicles 2018 (MISEV2018): A position statement of the International Society for Extracellular Vesicles and update of the MISEV2014 guidelines. J. Extracell. Vesicles.

[45] Silachev D., Goryunov K., Shpilyuk M., Beznoschenko O., Morozova N., Kraevaya E., Popkov V., Pevzner I., Zorova L., Evtushenko E. (2019). Effect of MSCs and MSC-Derived Extracellular Vesicles on Human Blood Coagulation. Cells.

[46] Varejão A.S.P., Melo-Pinto P., Meek M.F., Filipe V.M., Bulas-Cruz J. (2004). Methods for the experimental functional assessment of rat sciatic nerve regeneration. Neurol. Res..

[47] Bolte S., Cordelières F.P. (2006). A guided tour into subcellular colocalization analysis in light microscopy. J. Microsc..

